# A single-amino-acid mutation at position 225 in hemagglutinin attenuates H5N6 influenza virus in mice

**DOI:** 10.1080/22221751.2021.1997340

**Published:** 2021-11-07

**Authors:** Xingtian Kong, Lizheng Guan, Jianzhong Shi, Huihui Kong, Yaping Zhang, Xianying Zeng, Guobin Tian, Liling Liu, Chengjun Li, Yoshihiro Kawaoka, Guohua Deng, Hualan Chen

**Affiliations:** a College of Veterinary Medicine, Gansu Agricultural University, Lanzhou, People’s Republic of China; b State Key Laboratory of Veterinary Biotechnology, Harbin Veterinary Research Institute, Chinese Academy of Agricultural Sciences, Harbin, People’s Republic of China; c Division of Virology, Department of Microbiology and Immunology, Institute of Medical Science, University of Tokyo, Tokyo, Japan

**Keywords:** Influenza virus, H5N6 subtype, molecular basis, pathogenicity, mice

## Abstract

The highly pathogenic avian influenza H5N6 viruses are widely circulating in poultry and wild birds, and have caused 38 human infections including 21 deaths; however, the key genetic determinants of the pathogenicity of these viruses have yet to be fully investigated. Here, we characterized two H5N6 avian influenza viruses – A/duck/Guangdong/S1330/2016 (GD/330) and A/environment/Fujian/S1160/2016 (FJ/160) – that have similar viral genomes but differ markedly in their lethality in mice. GD/330 is highly pathogenic with a 50% mouse lethal dose (MLD_50_) of 2.5 log_10_ 50% egg infectious doses (EID_50_), whereas FJ/160 exhibits low pathogenicity with an MLD_50_ of 7.4 log_10_ EID_50_. We explored the molecular basis for the difference in virulence between these two viruses. By using reverse genetics, we created a series of reassortants and mutants in the GD/330 background and assessed their virulence in mice. We found that the HA gene of FJ/160 substantially attenuated the virulence of GD/330 and that the mutation of glycine (G) to tryptophan (W) at position 225 (H3 numbering) in HA played a key role in this function. We further found that the amino acid mutation G225W in HA decreased the acid and thermal stability and increased the pH of HA activation, thereby attenuating the H5N6 virus in mice. Our study thus identifies a novel molecular determinant in the HA protein and provides a new target for the development of live attenuated vaccines and antiviral drugs against H5 influenza viruses.

## Introduction

Influenza A virus is a single-stranded, negative-sense, segmented RNA virus of the family Orthomyxoviridae. Its genome harbours eight viral RNA (vRNA) segments encoding at least 17 proteins [1–6]. Influenza A viruses are categorized into 18 different HA subtypes and 11 different NA subtypes based on the different antigenicity of their two surface glycoproteins, hemagglutinin (HA) and neuraminidase (NA). All of these subtypes have been found in aquatic birds, which are viewed as the natural hosts of the viruses, with the exception of H17N10 and H18N11 subtypes, which were detected in bats [7,8]. Several subtypes of influenza viruses circulating in animals, including H5N1, H5N6, H7N9, H9N2, and H10N3, have shown clear threats to human health [9–15]. Understanding the pathogenesis of these potential pandemic viruses will lead to better pandemic preparedness.

Influenza viruses are prone to genetic mutations during replication, and sometimes one or two amino acid mutations can cause huge biological changes. For example, the mutation E627 K or D701N in PB2 can dramatically increase the virulence of avian influenza viruses in mammals [16,17]. The G622D mutation in PB1 can attenuate H5N1 avian influenza virus in mice by partially impairing the binding of PB1 to vRNA [18]. Several amino acid mutations in the PA protein are necessary for enhanced virulence of H5N1 viruses in mice or ducks [19,20]. Two amino acid mutations (A286 V and T437M) in NP have been shown to independently slow NP import to and export from the nucleus, thus impairing the viral life cycle and eliminating the virulence of H7N9 virus in mice [21], while asparagine at position 30, in combination with alanine at position 215, in the M1 protein can increase H5N1 virus virulence in mice [22]. Certain amino acid residues or regions of the NS1 protein enhance the virulence of H5N1 influenza viruses in chickens and mice by reducing the antiviral immune response of the host [23–25].

H5N6 viruses have been widely detected in wild birds and domestic poultry in many countries [26–28], and as of August 6, 2021, 38 human cases of infection with H5N6 viruses including 21 deaths have been reported to the WHO [29]. Here, we used two clade 2.3.4.4 H5N6 avian influenza viruses that are genetically similar but display considerably different virulence in mice as models to explore the genetic determinant(s) of their virulence difference. We identified a key amino acid in HA that contributes to the different pathogenicity between these two H5N6 viruses and explored the underlying mechanism.

## Materials and methods

### Ethics statement and facility

This study was performed in strict accordance with the recommendations in the Guide for the Care and Use of Laboratory Animals of the Ministry of Science and Technology of the People’s Republic of China. Studies with highly pathogenic H5N6 avian influenza viruses were carried out in a biosecurity level 3 laboratory approved for such use by the Chinese Ministry of Agriculture. The protocol was approved by the Committee on the Ethics of Animal Experiments of the Harbin Veterinary Research Institute (HVRI) of the Chinese Academy of Agricultural Sciences (CAAS).

### Cells and viruses

HEK293 T cells and Vero cells were cultured in Dulbecco’s modified Eagle’s medium (DMEM) supplemented with 10% fetal bovine serum. Human lung carcinoma (A549) cells were cultured in nutrient mixture F-12 Ham Kaighn’s modified (F-12 K) medium supplemented with 10% fetal bovine serum. All cells were cultured in 5% CO_2_ at 37°C. Two highly pathogenic H5N6 viruses containing RRRKR at the cleavage site in their HA, A/duck/Guangdong/S1330/2016 (GD/330) and A/environment/Fujian/S1160/2016 (FJ/160), were isolated from a duck and an environmental sample, respectively, collected in live poultry markets during our routine surveillance. The sequences of the two viruses are available in the Global Initiative on Sharing Avian Influenza Data database (accession numbers EPI1921655 to EPI1921670). Virus stocks were propagated in 10-day-old, specific-pathogen-free (SPF), embryonated chicken eggs and stored at −70°C until they were used.

### Construction of plasmids for virus rescue

An eight-plasmid reverse genetics system, reported previously [17], was used to create the reassortant viruses. We inserted the eight gene segments of FJ/160 and GD/330 into the vRNA-mRNA bidirectional transcription vector pBD [17] with a CloneExpress II One Step Cloning Kit (Vazyme, C112-02) to rescue FJ/160 and GD/330. Mutations were introduced into the HA gene of FJ/160 and GD/330 by means of site-directed mutagenesis (Invitrogen) according to the manufacturer’s protocol. The primer sequences used for the construction of the plasmids are shown in Table 1.

**Table 1.** Primers used for pBD cDNA construction and for introducing mutations into the HA genes of the mutant viruses.

Purpose	Primers (5′-3′)^a^	
	Forward	Reverse
PB2 amplification	TGCCGGCCAGCAAAAGCAGGTCAAATAT	CGGGTTATTAGTAGAAACAAGGTCGTTT
PB1 amplification	TGCCGGCCAGCAAAAGCAGGCAAACCAT	CGGGTTATTAGTAGAAACAAGGCATTT
PA amplification	TGCCGGCCAGCAAAAGCAGGTACTGAT	CGGGTTATTAGTAGAAACAAGGTACT
HA amplification	TGCCGGCCAGCAAAAGCAGGGGTTCAAT	CGGGTTATTAGTAGAAACAAGGGTGTTT
NP amplification	TGCCGGCCAGCAAAAGCAGGGTAGAT	CGGGTTATTAGTAGAAACAAGGGTAT
NA amplification	TGCCGGCCAGCAAAAGCAGGGTGAAA	CGGGTTATTAGTAGAAACAAGGGTGT
M amplification	TGCCGGCCAGCAAAAGCAGGTAGATGTT	CGGGTTATTAGTAGAAACAAGGTAGT
NS amplification	TGCCGGCCAGCAAAAGCAGGGTGACAA	CGGGTTATTAGTAGAAACAAGGGTGT
GD/330-HA-M214V mutation	TTAAACCAGAGATTG**G**TGCCAAAAATAGCTA	**C**CAATCTCTGGTTTAATGTTGATGTCCCAAC
GD/330-HA-G225W mutation	ACTAGATCCCAAGTAAAC**T**GGCAACAAGGAAG	**A**GTTTACTTGGGATCTAGTAGCTATTTTTGG
GD/330-HA-V537A mutation	CAATTTATTCAACAG**C**GGCGAGTTCCCTAGC	**G**CTGTTGAATAAATTGACAGTATTTGGTAAG

^a^ Nucleotides that were changed are underlined and in boldface.

## Virus rescue

293T cells in six-well plates were transfected with 4 μg of the eight plasmids by using Lipofectamine^TM^ LTX Reagent with PLUS^TM^ Reagent (Invitrogen) according to the manufacturer’s instructions. Six to eight hours later, the mixture was replaced with Opti-MEM (Gibco, Grand Island, NY, USA). After 48 h, the supernatants were harvested and injected into embryonated eggs for virus propagation. All of the rescued viruses were fully sequenced to ensure the absence of unwanted mutations.

### Studies with mice

Groups of eight 6-week-old female BALB/c mice (Beijing Experimental Animal Center, Beijing, China) were gently anesthetized with CO_2_ and inoculated intranasally with 10^6.0^ 50% egg infectious doses (EID_50_) of H5N6 influenza virus in a volume of 50 µL. On day 3 post-inoculation (p.i.), three of the eight mice were euthanized, and their nasal turbinate, lungs, brain, kidneys, and spleen were collected and titrated for virus infectivity in eggs. The remaining mice in each group were monitored daily for weight loss and mortality for 14 days. The 50% mouse lethal dose (MLD_50_) was determined by inoculating groups of five mice with 10-fold serial dilutions containing 10^1.0^–10^6.0^ or 10^7.0^ EID_50_ of virus in a volume of 50 µL. The results were calculated by using the method of Reed and Muench [30].

### Viral growth curve

A549 cells were grown on 12-well plates and inoculated with viruses at a multiplicity of infection (MOI) of 0.01. The inoculum was removed after incubation at 37°C for 1 h. Then the cells were washed three times with phosphate-buffered saline (PBS) and were maintained in Opti-MEM at 37°C. The virus-containing culture supernatants were collected from triplicate cultures at 12, 24, and 48 h post-infection (p.i.), and virus titers were determined in eggs by using the method of Reed and Muench [30].

### Syncytium formation assay

A previously described syncytia formation assay with slight modification was used to determine the pH of HA activation [31,32]. Briefly, Vero cells were grown in 6-well plates and infected with viruses at an MOI of 3. At 16 h post-infection, the cells were treated with 5 μg/mL^−1^ of TPCK trypsin for 15 min and incubated in prewarmed pH-adjusted PBS (pH 4.8–6.2, increasing by increments of 0.1). Next, the low-pH PBS was replaced with DMEM containing 10% FBS, and the cells were incubated for 3 h at 37 °C to allow for syncytium formation. After 3 h, the cells were fixed with 4% paraformaldehyde and stained with mouse anti-NP primary antibody and goat anti-mouse IgG (H + L) Alexa Fluor Plus 488 secondary antibody (Thermo Fisher Scientific). Images were taken on the Evos XL cell imaging system (Life Technologies). To quantify syncytium formation, cell nuclei were counted in five randomly chosen fields and the highest pH at which syncytium formation was recorded.

### Acid stability assay

The acid stability test was performed as previously described with slight modifications [33,34]. Briefly, viruses (10^6.0^ EID_50_ in PBS) were mixed with different pH-adjusted PBS buffers and incubated at 37°C for 1 h. The virus titers were then determined by measuring EID_50_.

### Thermostability assay

The thermostability test was performed as previously described with slight modifications [12,31]. In brief, viruses (10^6.0^ EID_50_ in PBS) were incubated for up to 6 h at 50°C. The virus titers were determined hourly by measuring EID_50_.

## Structure prediction of the HA protein of the H5N6 virus

Three-dimensional (3-D) structure of the HA protein was predicted by using I-TASSER algorithm [35].

### Statistical analysis

Statistical significance between different groups was performed by using the multiple *t* tests in GraphPad Prism 8 software. *P *< 0.05 was considered statistically significant.

## Results

### HA gene of FJ/160 virus attenuates the GD/330 virus in mice

The replication and virulence of these two viruses GD/330 and FJ/160 were evaluated in BALB/c mice. GD/330 replicated efficiently in the nasal turbinate, lungs, spleen, kidneys, and brain of mice, with an MLD_50_ of 2.5 log_10_ EID_50_ (Table 2), whereas FJ/160 replicated in the nasal turbinate, lungs, and spleen, but not in the kidneys or brain of mice, yielding an MLD_50_ of 7.4 log_10_ EID_50_ (Table 2).

**Table 2.** Replication and lethality of H5N6 viruses in mice^a^.

Virus	Virus titer (log_10_ EID_50_/mL), mean ± SD^b^					MLD_50_ (Log_10_ EID_50_) ^c^
	Nasal Turbinate	Lung	Spleen	Kidney	Brain	
GD/330	5.8 ± 0.4	7.1 ± 0.3	3.4 ± 0.1	3.6 ± 0.1	2.5 ± 0.3	2.5
rRD-330	4.9 ± 0.5	7.5 ± 0.0	3.3 ± 0.1	3.2 ± 0.4	2.3 ± 0.5	2.5
FJ/160	3.1 ± 0.6	6.5 ± 0.3	1.1 ± 0.5	-	-	7.4
rFJ-160	2.3 ± 0.5	5.4 ± 0.1	0.8 ± 0.4	-	-	7.4

^a^ Six-week-old female BALB/c mice were used for these studies.

^b^ Groups of three mice were inoculated intranasally with 10^6.0^ EID_50_ of the test virus in a 50-µL volume and were killed on day 3 postinoculation (p.i.); organs were then collected for virus titration in eggs. -, no virus was detected in undiluted samples. Virus titers of mice were compared by using the Student-Newman-Keuls test. SD, standard deviation.

^c^ The 50% mouse lethal dose (MLD_50_) was determined by intranasally inoculating groups of five mice with 10-fold serial dilutions containing 10^1.0^–10^6.0^ EID_50_ of virus in a 50-µL volume.

**Figure 1.**
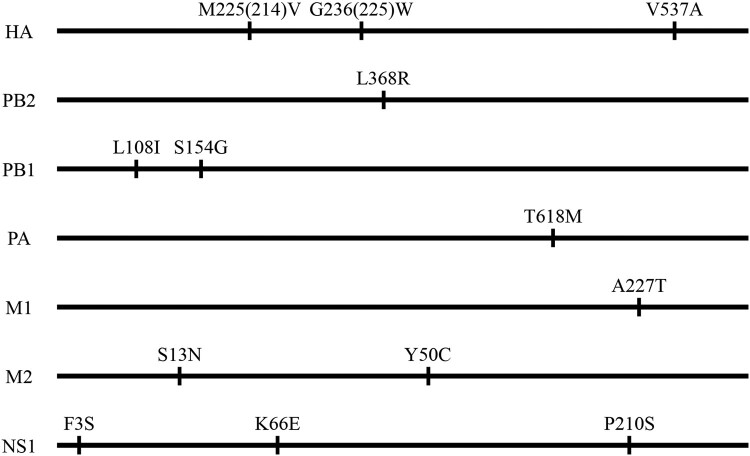
Amino acid differences between the two H5N6 avian influenza viruses. The amino acid differences between GD/330 and FJ/160 are shown as single letters at the indicated positions. Each amino acid of GD/330 is shown before the number of the position, and each amino acid of FJ/160 is shown after the number of the position. The amino acid positions in HA1 for H3 numbering are shown in brackets.

**Figure 2.**
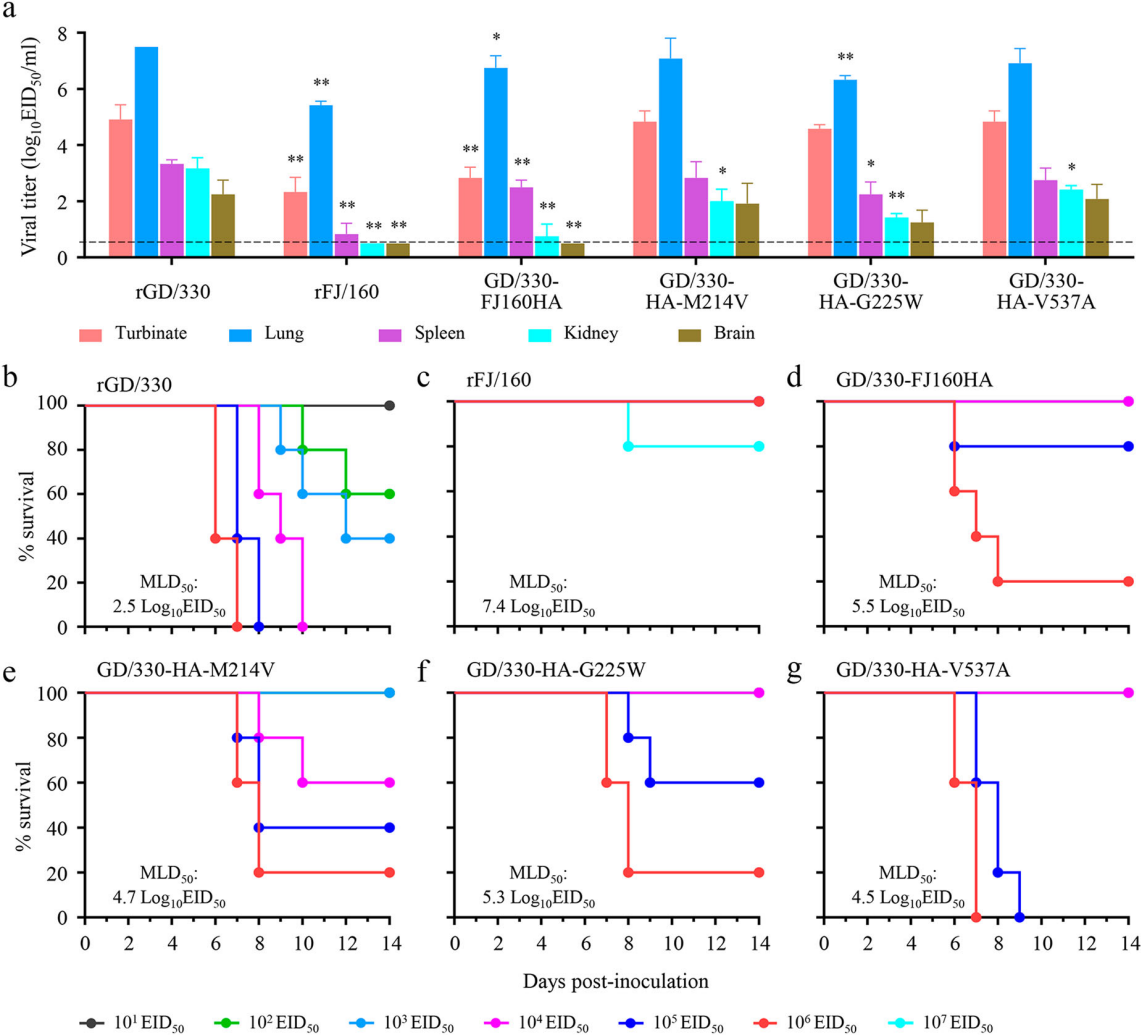
Replication and lethality of H5N6 avian influenza viruses in BALB/c mice. (a) Virus titers in organs of mice inoculated intranasally with 10^6.0^ EID_50_ of different H5N6 viruses. Organs were collected on day 3 post-inoculation for virus titration in eggs. Data are means ± standard deviations (SD). The dashed lines indicate the lower limit of virus detection. The statistical analysis was conducted by using multiple *t* tests with GraphPad Prism 8 software. *, *P<*0.05 compared with the virus titers in the corresponding organs of rGD/330 virus-infected mice. **, *P<*0.01 compared with the virus titers in the corresponding organs of rGD/330 virus-infected mice. (b–g) MLD_50_ for mice infected with each indicated virus.

Sequence analysis indicated that the two viruses differ by only 13 amino acids across seven proteins: HA, PB2, PB1, PA, M1, M2, and NS1 (Figure 1). To explore which gene(s) may have contributed to the difference in pathogenicity between the two viruses in mice, we first established an eight-plasmid reverse genetics system and rescued the two viruses by using a strategy we reported previously [17]. Mouse studies demonstrated that the rescued viruses rGD/330 and rFJ/160 retained similar replication and pathotypes as their wild-type parental viruses (Table 2, Figure 2(a–c)).

To avoid any “gain-of-function” concerns involving experiments that may increase the pathogenicity of potential pandemic pathogens, we selected GD/330 as the backbone to generate six reassortants by reverse genetics, each bearing one gene from FJ/160 and the remaining segments from GD/330 (designated GD/330-FJ160PB2, GD/330-FJ160PB1, GD/330-FJ160PA, GD/330-FJ160HA, GD/330-FJ160M, and GD/330-FJ160NS, respectively), and evaluated their replication and pathogenicity in mice. The virulence of the reassortants that carried the PB2, PB1, PA, M, or NS gene of FJ/160 in the GD/330 background was similar to that of the rGD/330 virus (MLD_50_, 2.5 log_10_ EID_50_) (Figure S1). However, the virus carrying the HA gene of FJ/160 (GD/330-FJ160HA) was substantially attenuated by 1000-fold in mice compared with rGD/330 (MLD_50_, 2.5 versus 5.5 log_10_ EID_50_); moreover, it was not detected in the brain of mice, and the titers in the other mouse organs infected with GD/330-FJ160HA were lower than those in the corresponding organs of mice infected with rGD/330 (Figure 2(d)). These results demonstrate that HA plays an essential role in the virulence of GD/330 in mice.

### Glycine at position 225 in HA plays an essential role in the virulence of GD/330 in mice

There are only three amino acids differences, at positions 214, 225 (since these two amino acids are located in the HA1, H3 numbering is used for these two sites throughout the manuscript), and 537, respectively, in the HA gene between FJ/160 and GD/330 (Figure 1). To pinpoint which amino acid(s) in HA determine(s) the virulence of GD/330 in mice, we generated three mutant viruses – GD/330-HA-M214 V, GD/330-HA-G225W, and GD/330-HA-V537A – and tested their pathogenicity in mice. We detected all three mutants in all five tested organs of mice infected with these three mutants; however, the titers in the spleens or kidneys of the mice infected with the mutants were markedly lower than those in the corresponding organs of mice infected with rGD/330 (Figure 2(a)). The MLD_50s_ of GD/330-HA-M214 V, GD/330-HA-G225W, and GD/330-HA-V537A were 4.7 log_10_ EID_50_, 5.3 log_10_ EID_50_, and 4.5 log_10_ EID_50_, respectively, and their virulence was reduced by 160-fold, 630-fold, and 100-fold, respectively, compared with that of rGD/330 (Figure 2(e–g)). These results indicate that the substitution of glycine with tryptophan at position 225 in HA played an important role in attenuating the virulence of GD/330 in mice.

### The mutation G225W in HA impairs the replication of GD/330 in A549 cells

**Figure 3.**
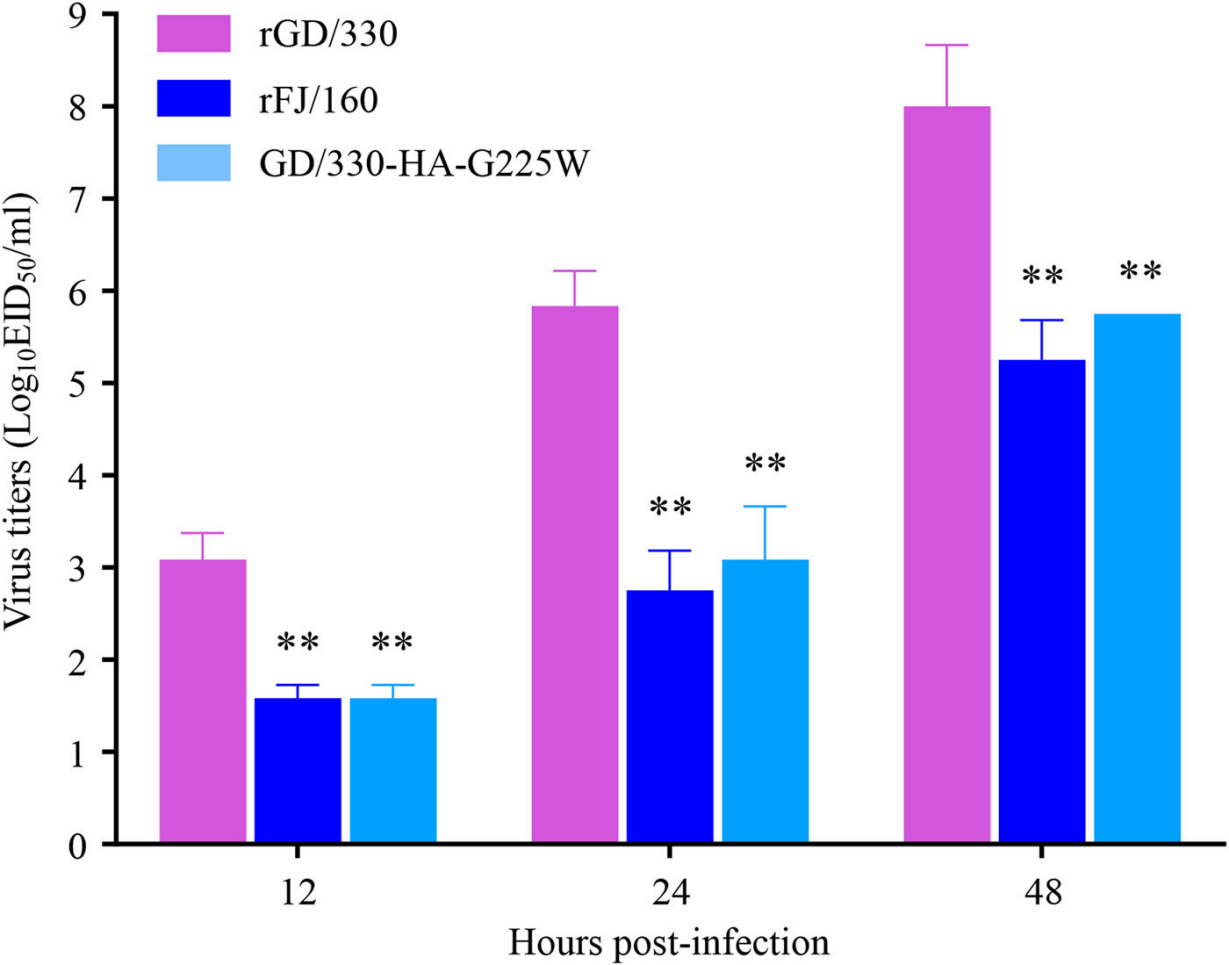
Multicycle replication of H5N6 avian influenza viruses in A549 cells. A549 cells were infected with three viruses at an MOI of 0.01, and the supernatants were collected at the indicated times and titrated in eggs. The data shown are the means of three replicates; the error bars indicate standard deviations. The statistical analysis was conducted by using multiple *t* tests. *, *P<*0.05 compared with the virus titers of rGD/330 virus-infected cells. **, *P<*0.01 compared with the virus titers of rGD/330 virus-infected cells.

Replication efficiency of influenza virus is correlated to its virulence [36,37]. To investigate whether the G225W mutation affects viral replication, we evaluated the replication of rGD/330, rFJ/160, and GD/330-HA-G225W in A549 cells. A549 cells were infected with the viruses at an MOI of 0.01, and the supernatants were collected at different timepoints for viral titration in eggs. We found that the rGD/330 replicated more efficiently than the rFJ/160 and GD/330-HA-G225W, and the titers of rGD/330 were significantly higher than those of rFJ/160 and GD/330-HA-G225W at all three timepoints post infection, whereas the viral titers of rFJ/160 and GD/330-HA-G225W were comparable (Figure 3). These results indicated that the amino acid mutation of G225W in HA impaired the replication of H5N6 virus in mammalian cells.

### The mutation G225W in HA increases the pH of HA activation

**Figure 4.**
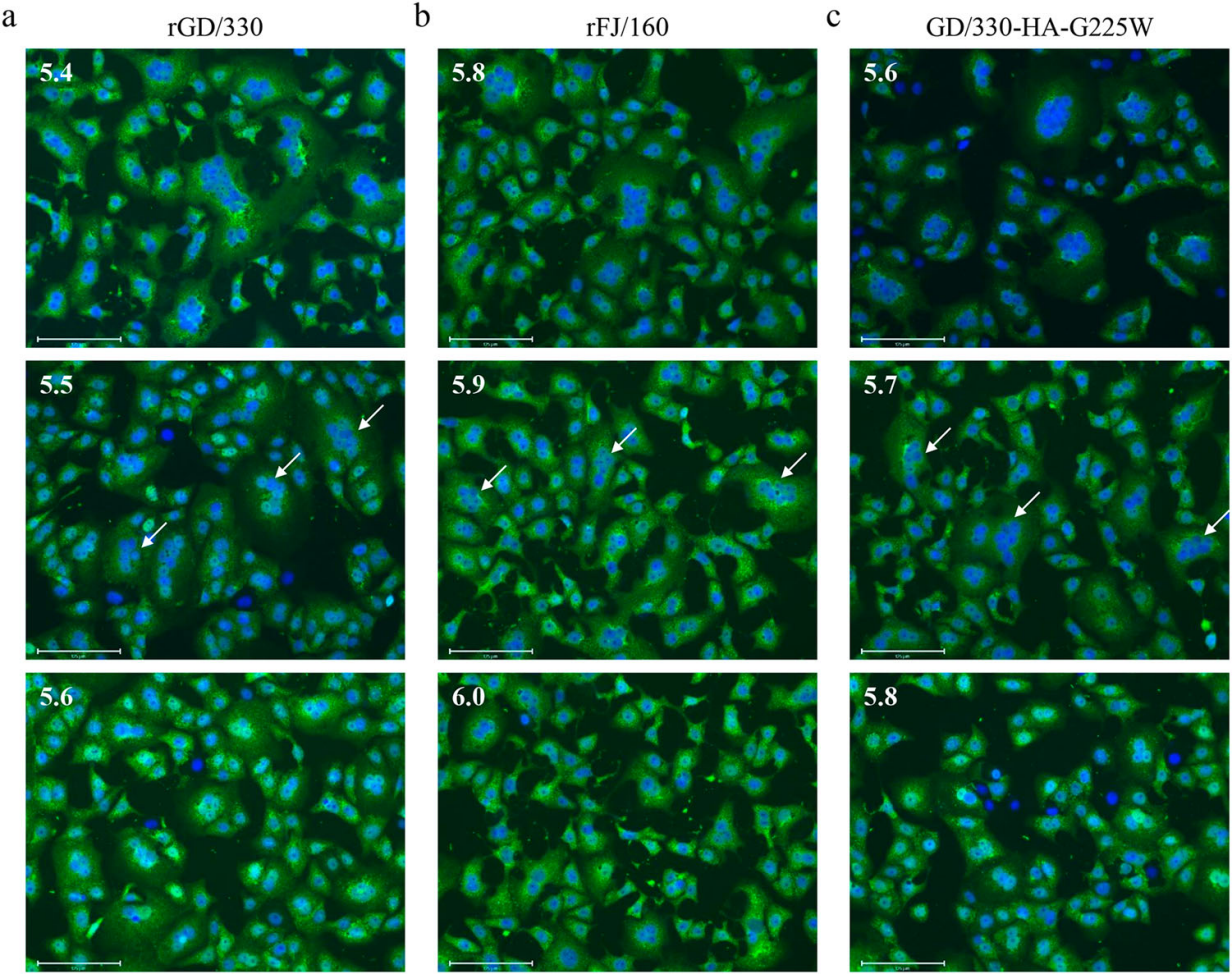
The effect of pH on HA activation of different H5N6 viruses. Vero cells infected with (a) rGD/30, (b) rFJ/160, or (c) GD/330-HA-G225W at an MOI of 3 were incubated with pH-adjusted PBS (4.8–6.2). The highest pH at which syncytia formed (arrow) above 50% was defined as the pH threshold.

HA is triggered by low pH to undergo irreversible conformational changes that facilitate membrane fusion between the virus envelope and the endosomal membrane [38]. Several studies have reported that the lower the pH of HA activation, the more efficiently the influenza virus replicates and the more virulent it is to mammals and humans [33,34]. Therefore, we investigated the pH of HA activation of rGD/330, rFJ/160, and GD/330-HA-G225W by using a syncytium formation assay in Vero cells [31,32]. We found that the pH of HA activation of rGD/330, rFJ/160, and GD/330-HA-G225W was 5.5, 5.9, and 5.7, respectively (Figure 4). These results indicate that the G225W mutation in HA increases the pH of HA activation, which, in turn, affects the replication in cells and pathogenicity of H5N6 virus in mice.

### The amino acid at position 225 in HA affects viral acid and thermal stability

**Figure 5.**
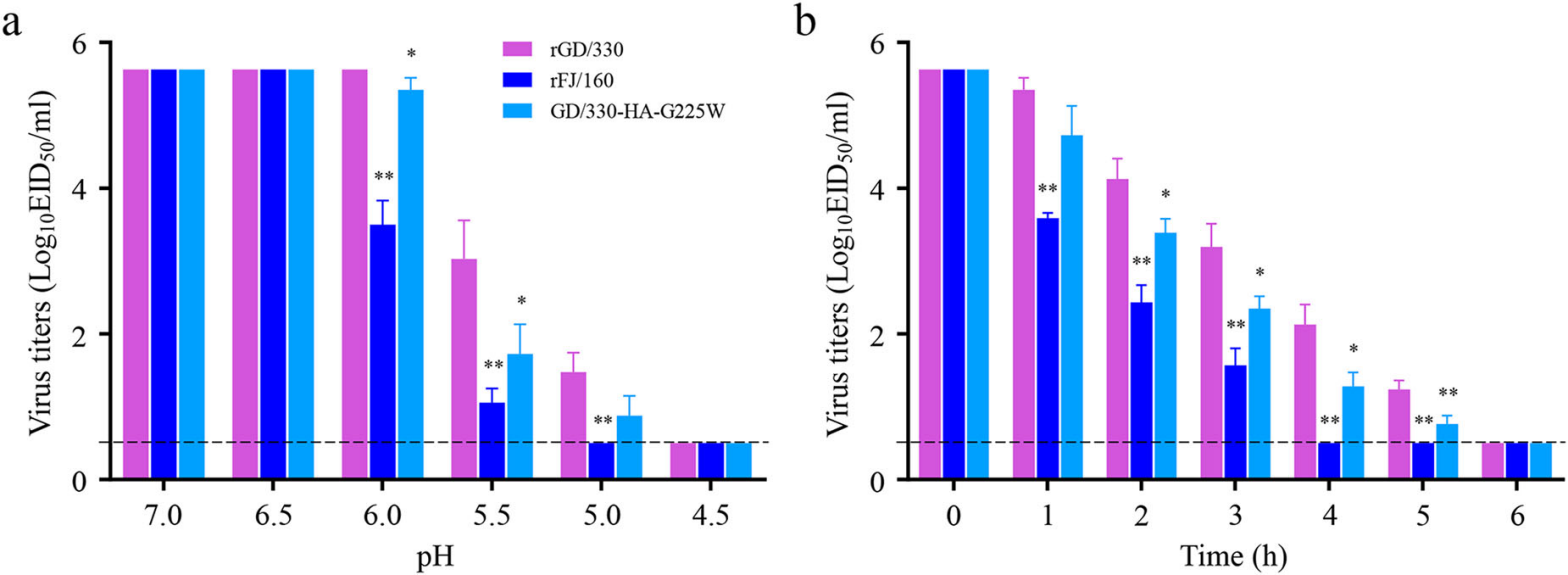
The stability of H5N6 avian influenza viruses. (a) Acid stability of three H5N6 viruses. 10^6.0^ EID_50_ of each virus was diluted in PBS adjusted to the indicated pH and incubated at 37°C for 1 h. Virus titers were then determined in eggs. (b) Heat stability of three H5N6 viruses. 10^6.0^ EID_50_ of each virus was incubated at 50°C for 6 h, and virus titers were then determined in eggs hourly. The statistical analysis was conducted by using multiple *t* tests with GraphPad Prism 8 software. *, *P<*0.05 compared with the titers of rGD/330 virus. **, *P<*0.01 compared with the titers of rGD/330 virus.

The acid and thermal stability of influenza viruses correlates with their pathogenicity in mammals [39,40]. To investigate whether G225W in HA affects acid stability, we incubated 10^6.0^ EID_50_ of rGD/330, rFJ/160, and GD/330-HA-G225W in PBS with different pH values at 37°C for 1 h and then titrated the viruses in chicken embryos. The titers of the three viruses were similar after being treated with PBS at pH 7.0 or 6.5, and no live virus was detected after being treated with PBS at pH 4.5. However, after treatment with PBS at pH 6.0 and 5.5, the three viruses showed significant differences, with the titers of rGD/330 being the highest, followed by GD/330-HA-G225W and rFJ/160 (Figure 5(a)). To investigate whether G225W in HA affects thermal stability, we incubated 10^6.0^ EID_50_/0.1 mL of each virus at 50°C for 6 h in total, evaluating the viral titers after every hour of incubation. We found that the titers of all three viruses declined over time, but rFJ/160 declined faster than the others and completely lost its infectivity after four hours of incubation. Although rGD/330 and GD/330-HA-G225W lost their infectivity after six hours of incubation, the titers of GD/330-HA-G225W were significantly lower than those of rGD/330 at four time points (2, 3, 4 and 5 h) (Figure 5(b)). These results indicate that the G225W mutation in HA reduces the acid and thermal stability of H5N6 influenza viruses, which may contribute to the decreased virulence of GD/330-HA-G225W in mice.

### The G225W mutation increases the steric hindrance in the HA structure

**Figure 6.**
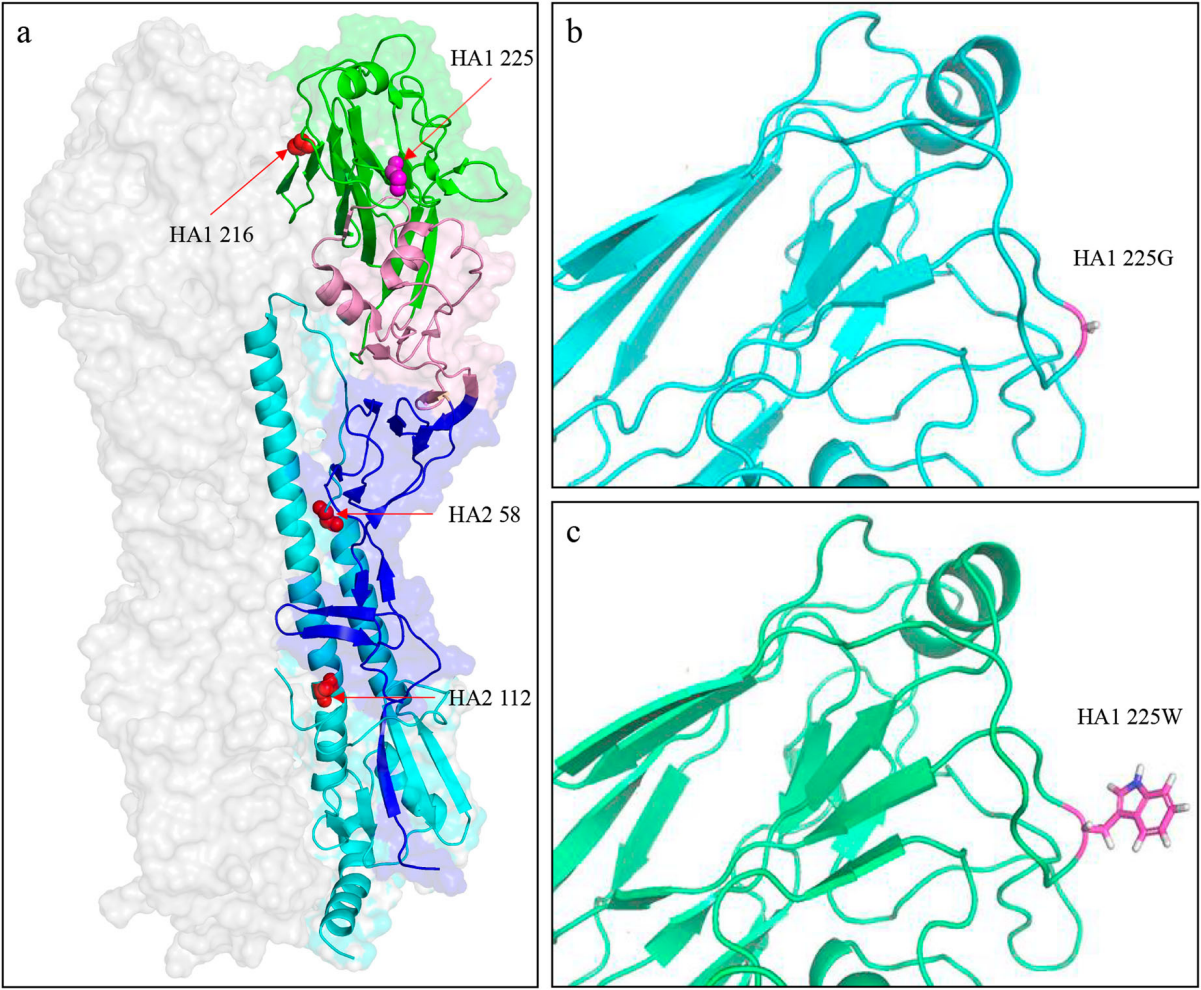
Three-dimensional (3D) structures and analysis of the HA protein of H5N6 viruses. (a) Positions affecting the pH of HA activation are displayed in the structure (PDB ID: 5HUF). The domains of the HA monomer are marked with different colours: receptor-binding domain (green), vestigial esterase subdomain (pink), and N- and C-terminal segments of HA1 (F′ fusion subdomain, blue), and HA2 (cyan). The positions of residues that affect the pH activation are shown as spheres. The 3D structures of HA of GD330 (b) and GD330-HA-G225W (c) were predicted by using I-TASSER algorithm, and the amino acid at position 225 was shown as stick in the structural illustration.

To further understand the molecular basis of G225W mutation in the HA of the H5N6 virus, we predicted the 3D structures of the HA protein of GD/330 and GD/330-HA-G225W by using I-TASSER algorithm [35]. The amino acid at position 225 is located at the membrane distal end of the HA monomer (Figure 6(a)). The G225W mutation did not change the hydrogen bonds or salt bridges in the HA according to the structural analysis; However, the side chains of the amino acids G and W are different: the smallest amino acid G has a single hydrogen atom as its side chain (Figure 6(b)), while the largest amino acid W has an indole as its side chain (Figure 6(c)). We therefore speculate that the steric hindrance may have contributed to the observed functional difference of HA in the GD/330 and GD/330-HA-G225W viruses.

## Discussion

In the present study, we isolated two H5N6 avian influenza viruses – FJ/160 and GD/330 – that are genetically similar but show strikingly different pathogenicity in mice. By using reverse genetics, we found that the HA gene plays a key role in the virulence difference between these two viruses in mice and that the mutation G225W in HA significantly attenuates the virulence of GD/330 in mice. We further demonstrated that the G225W mutation in HA increases the pH of HA activation and reduces the acid and thermal stability of the H5N6 virus, thereby attenuating the virulence of the H5N6 virus in mice.

HA is a multifunctional protein that affects the biological characteristics of influenza viruses in many ways[12,16,41,42]. HA is critical in determining the pathogenicity of various subtypes of influenza viruses. The motifs of multiple basic amino acids in the cleavage site of HA play principal roles in the virulence of highly pathogenic H5 and H7 avian influenza viruses in chickens and mice [12,16]. Zhao et al. demonstrated that the substitution G158N introduces an N-linked glycosylation site at positions 158–160 of the HA protein and that this N-linked glycosylation enhances viral productivity in infected mammalian cells and exacerbates host immune and inflammatory responses to viral infection [42]. In the present study, we found that the G225W mutation in HA attenuates the pathogenicity of H5N6 viruses in mice by reducing the acid and thermal stability of the virus and increasing the pH of HA activation. These findings confirm that different genetic changes in the HA protein of influenza virus alter its pathogenicity through different molecular mechanisms.

HA is also responsible for receptor-binding and plays an important role in the transmission of influenza virus [43–47]. The amino acid mutation G225D at position 225 in HA was shown to alter the receptor-binding preference and abolish the transmission of the 1918/H1N1 virus in ferrets [48], and the mutation E225G in HA abolishes the transmission of Eurasian avian-like H1N1 swine influenza virus in guinea pigs [49]. We analysed 7558 H5 viruses and found that 7548 viruses have a 225G in their HA, and only one virus (FJ/160) has a 225W in its HA, indicating that 225G is highly conserved in H5 viruses. The mutation G225W in HA attenuates the virulence of H5N6 virus in mice; how this mutation affects the receptor-binding preference and transmissibility of the H5N6 virus in mammals remains to be investigated.

Previous studies have identified several mutations at different positions in the HA of different influenza viruses that reduce the pH of HA activation and enhance the replication and virulence of these viruses in mammals [39]. Some of these mutations, such as HA2 K58I, HA2 D112G, HA1 K216E, were reported to lead the creation or destruction of hydrogen bonds or salt bridges in HA [40]. In the present study, we found that the mutation G225W in HA1 increases the pH of HA activation and attenuates the H5N6 virus GD/330 in mice, but this G225W mutation in HA does not affect any hydrogen bonds or salt bridges. Therefore, the pH of HA activation can be affected by multiple amino acids in both HA1 and HA2 through different mechanisms.

In summary, we found that the mutation G225W in HA plays a key role in attenuating the H5N6 virus in mice by increasing its pH of HA activation and decreasing its acid and thermal stability. Our findings emphasize how the HA gene is an essential pathogenic determinant of the influenza viruses and demonstrate that this novel virulence-related signature in HA may provide a new target for the development of attenuated vaccines and antiviral drugs.

## Supplementary Material

Supplemental Material
