# Supplementary figures and images for: A single-amino-acid mutation at position 225 in hemagglutinin attenuates H5N6 influenza virus in mice
Source: Emerg Microbes Infect. 2021 Nov 7;10(1):2052–61. doi: 10.1080/22221751.2021.1997340 (PMC8583753; doi:10.1080/22221751.2021.1997340)

**Supplementary file**

Figure S1


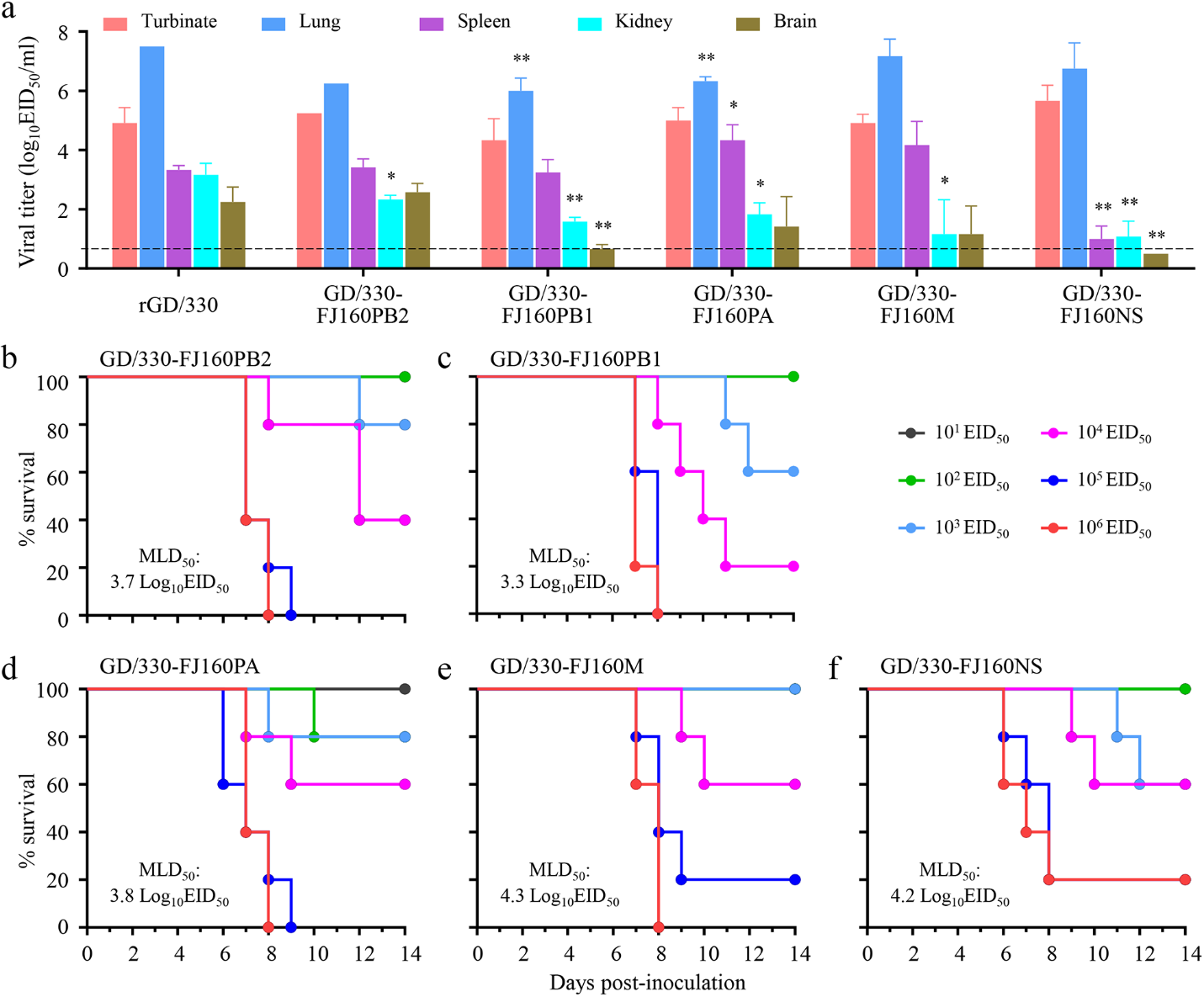

Supplement: Supplemental Material [file TEMI_A_1997340_SM6230.docx]
